# Supplementary material for: Content-rich biological network constructed by mining PubMed abstracts
Source: BMC Bioinformatics. 2004 Oct 8;5:147. doi: 10.1186/1471-2105-5-147 (PMC528731; doi:10.1186/1471-2105-5-147)
Supplement: Additional File 2 — The original results of the above study (non-essential files are deleted to keep the file size under the limit set by BMC bioinformatics). [file 1471-2105-5-147-S2.bz2 › chilibotAdditionalFile2/dip50/444ID9468473E10421/html/CCL3_CCR5.html]

 


 **CCL3** and **CCR5** 
  
Found 94 abstracts in PubMed, retrieved 50.  
 

 What does Google say? 
 PDF only 
| .edu only 

---

**Interactive relationship** (e.g. stimulation, inhibition, etc)

**Inhibitory relationship**- Supernatants from samples with high copy number are able to more potently chemoattract  **CCR5**  expressing cells, an effect blocked with anti  **CCL3**   **CCL3**  L1 antibodies.  Ref: 12355456 Eur J Immunol, 2002
- Whereas mutation of residues in ECL2 of  **CCR5**  dramatically reduced the binding of RANTES and MIP 1 alpha  [ **CCL3** ]  and their ability to induce signaling, interaction with vMIP II was not altered by any mutation in the exoloops of the receptor.  Ref: 11700073 J Mol Biol, 2001
- The MIP 1 alpha  [ **CCL3** ]  isoform LD78 beta, that was purified form natural sources, inhibited HIV infection completely in  **CCR5**  transfected cells, mononuclear leukocytes and purified monocytes at low ng ml concentrations.  Ref: 12649932 Verh K Acad Geneeskd Belg, 2002
- Several potential mechanisms for expansion of HIV 1 in situ were found, including augmentation in expression of tumor necrosis factor TNF alpha and the HIV 1 noninhibitory beta chemokine MCP 1, low presence of HIV 1 inhibitory beta chemokines MIP 1 alpha  [ **CCL3** ] , MIP 1 beta, and RANTES regulated on activation, normal T expressed and secreted, and upregulation in expression of the HIV 1 coreceptor,  **CCR5** , by pleural fluid mononuclear cells.  Ref: 11579270 J Acquir Immune Defic Syndr, 2001
**Neutral relationship**- The CC chemokine receptor 5  [ **CCR5** ]   **CCR5**  binds the chemokine ligands RANTES CCL5 and MIP 1alpha  **CCL3** , which have been implicated in the development of alveolitis in sarcoidosis.  Ref: 11803051 Immunol Lett, 2002
- An abortive ligand induced activation of CCR1 mediated downstream signaling event and a deficiency of  **CCR5**  expression are associated with the hyporesponsiveness of human naive CD4 T cells to  **CCL3**  and CCL5.  Ref: 12055240 J Immunol, 2002
- Strong interaction with  **CCR5**  means that  **CCL3**  L1, and particularly its 2 variant, are by far the most potent natural HIV entry inhibitors described to date.  Ref: 12355456 Eur J Immunol, 2002
- A  **CCR5**  chimera with the distal N ter residues of CXCR2 bound MIP 1 alpha  [ **CCL3** ]  and vMIP II with an affinity similar to that of the wild type receptor.  Ref: 11700073 J Mol Biol, 2001
- Although virus specific CD8 T cells from  **CCL3**  mice were CD44 high, they remained CD62L high and CD25 low, retained CCR7 expression, and contained limited transcripts of the proinflammatory chemokine receptors  **CCR5**  and CXCR3 compared with virus specific CD8 T cells from  **CCL3**  mice.  Ref: 12634360 J Virol, 2003
- However, the remaining expression of CCR1 and  **CCR5**  correlated reciprocally with an ongoing unresponsiveness to the CCR1 and  **CCR5**  agonists  **CCL3**  macrophage inflammatory protein 1alpha MIP 1alpha, CCL4 MIP 1beta, and CCL5 regulated on activation, normal T expressed and secreted RANTES, all chemokines binding to these two receptors.  Ref: 12885942 J Leukoc Biol, 2003
- We and others have shown that the human beta chemokine  **CCL3**  L1, unlike its related non allelic isoform  **CCL3** , has high affinity for the chemokine receptors D6, CCR3 and  **CCR5** .  Ref: 12355456 Eur J Immunol, 2002
- High levels of  **CCL3**  and CCL4 were produced within 8 h after infection, which is likely to account for the observed  **CCR5**  down modulation on Mtb infected DC.  Ref: 12538673 J Immunol, 2003
- **CCR5**  is a G protein coupled receptor responding to four natural agonists, the chemokines RANTES regulated on activation normal T cell expressed and secreted, macrophage inflammatory protein MIP 1 alpha  [ **CCL3** ] , MIP 1 beta, and monocyte chemotactic protein MCP 2, and is the main co receptor for the macrophage tropic human immunodeficiency virus strains.  Ref: 12411445 J Biol Chem, 2003
- Our studies showed that LD infection caused the upregulation of three beta chemokines macrophage inflammatory protein 1 alpha  [ **CCL3** ]  MIP 1alpha, MIP 1beta and RANTES regulated on activation normal T cell expressed and secreted, one alpha chemokine interleukin 8 IL 8 and the CC chemokine receptor 5  [ **CCR5** ]   **CCR5**  but not CCR1, as evident from reverse transcriptase polymerase chain reaction RT PCR analysis.  Ref: 12662300 Scand J Immunol, 2003
- Survival of  **CCR5**  mice was equivalent to that of controls, indicating that the enhanced susceptibility of  **CCL3**  mice to K. pneumoniae is mediated via another  **CCL3**  receptor, presumably CCR1.  Ref: 11553580 Infect Immun, 2001
- Moreover,  **CCL3**  L1, but not  **CCL3** , is susceptible to cleavage by CD26, creating a truncated 2 form with enhanced affinity for CCR1 and  **CCR5** .  Ref: 12355456 Eur J Immunol, 2002
- The higher RANTES production by MS gamma delta T cell lines, together with a lower expression of  **CCR5** , may reflect an autoregulatory loop, caused by an increased production of its ligands RANTES, MIP 1 alpha  [ **CCL3** ] , and MIP 1 beta or due to other pro inflammatory cytokines.  Ref: 12173306 Clin Immunol, 2002

**Non-interactive relationship** (e.g. studied together, co-existance, homology, etc.)

- The  **CCR5**  ligands, RANTES and MIP 1 alpha  [ **CCL3** ] , are expressed in the spinal cords of mice at EAE onset.  Ref: 12667654 J Neuroimmunol, 2003
- CONCLUSIONS We conclude that the phosphorylation of p38 MAPK associated with the activation of NF kappa B may be involved in the upregulation of intrarenal MIP 1 alpha  [ **CCL3** ]  and the utilization of  **CCR5**  signalling, which may result in human crescentic glomerulonephritis.  Ref: 12032188 Nephrol Dial Transplant, 2002
- Stimulation of these  **CCR5**  positive T cells with the relevant chemokine MIP 1 alpha  [ **CCL3** ]  elicited Ca 2 influx, showing that IL 12 induced  **CCR5**  is functional.  Ref: 11500825 Eur J Immunol, 2001
- The expression of the chemokines macrophage inflammatory protein 1 alpha  [ **CCL3** ]  MIP 1alpha and interferon gamma inducible protein 10 IP 10 and of their respective receptors,  **CCR5**  and CXCR3, were more prevalent and higher in aggressive periodontitis, and associated with higher interferon gamma IFN gamma expression and lower interleukin 10 IL 10 expression.  Ref: 12608917 J Periodontal Res, 2003
- Characterization of 15  **CCR5**  alanine scanning mutants of charged extracellular amino acids revealed that alteration of acidic residues in the distal N ter abrogated binding of RANTES, MIP 1 alpha  [ **CCL3** ] , and vMIP II.  Ref: 11700073 J Mol Biol, 2001
- RANTES, MIP 1 alpha  [ **CCL3** ] , and vMIP II bound  **CCR5**  at high affinity, but demonstrated partial cross competition.  Ref: 11700073 J Mol Biol, 2001
- Molecular anatomy of  **CCR5**  engagement by physiologic and viral chemokines and HIV 1 envelope glycoproteins differences in primary structural requirements for RANTES, MIP 1 alpha  [ **CCL3** ] , and vMIP II Binding.  Ref: 11700073 J Mol Biol, 2001
- In parallel, CCR1 and  **CCR5**  expression is down regulated and CXCL8, CCL2,  **CCL3**  and CCL5 production is up regulated.  Ref: 12548563 Eur J Immunol, 2003
- In contrast, the loss of the  **CCR5**  ligand,  **CCL3** , led to an early delay in tumor growth that did not persist, while the absence of the  **CCR5**  ligand, CCL5, had no effect.  Ref: 12682253 J Immunol, 2003
- Furthermore, urinary levels of MIP 1 alpha  [ **CCL3** ] , the cognate ligand for  **CCR5** , were determined by enzyme linked immunosorbent assay.  Ref: 12032188 Nephrol Dial Transplant, 2002
- In addition the beta chemokines RANTES regulated on activation T cell expressed and secreted, macrophage inflammatory protein 1 alpha  [ **CCL3** ]  MIP 1alpha, and MIP 1beta can block HIV 1 entry into cells by preventing the binding of the macrophage trophic HIV 1 strains to the coreceptor  **CCR5** .  Ref: 11495560 Infect Dis Obstet Gynecol, 2001
- Engagement of  **CCR5**  by vMIP II, but not RANTES or MIP 1 alpha  [ **CCL3** ]  blocked the binding of monoclonal antibodies to the receptor, providing additional evidence for a distinct mechanism for viral chemokine binding.  Ref: 11700073 J Mol Biol, 2001
- After 2 to 4 weeks, L. amazonensis infected mice had significantly delayed and depressed expression of inflammatory cytokines interleukin 12 IL 12, gamma interferon, IL 1alpha, IL 1beta, CC chemokines CC chemokine ligand 3  [ **CCL3** ]   **CCL3**  macrophage inflammatory protein 1alpha MIP 1alpha, CCL4 MIP 1beta, CCL5 RANTES, MIP 2, and chemokine receptors CCR1, CCR2,  **CCR5**  in foot tissues and draining lymph nodes compared to the expression in L. major infected controls.  Ref: 12874303 Infect Immun, 2003
- Using self organizing maps, we identified nine genes CD4, CCR3,  **CCR5** , LT beta, MIP 1 alpha  [ **CCL3** ] , MIP 2, CD8 alpha, IP 10, and RANTES, each with a unique profile of transcriptional expression, that reproduce the differentiation of states of rejection in dendrograms.  Ref: 11818457 J Leukoc Biol, 2002
- Thus, a failure of the ligand induced activation of CCR1 mediated downstream signaling event as well as a deficiency of  **CCR5**  expression may be involved in the hyporesponsiveness of naive CD4 T cells to  **CCL3**  and CCL5.  Ref: 12055240 J Immunol, 2002
- To test the in vivo relevance of this, we used a murine melanoma system and knockout mice to investigate the function of the chemokine receptor  **CCR5**  and its ligands, CCR ligand CCL 3  [ **CCL3** ]  and CCL5.  Ref: 12682253 J Immunol, 2003
- Since both human MIP 1alpha subtypes LD78alpha  [ **CCL3** ]  and LD78beta and MIP 1beta signal through  **CCR5** , the major co receptor for M tropic HIV 1 strains, these chemokines are capable of inhibiting HIV 1 infection in susceptible cells.  Ref: 12401480 Cytokine Growth Factor Rev, 2002
- Bacterial LPS protects primary human macrophages from infection by  **CCR5**  tropic HIV 1 isolates through the release of the CC chemokines RANTES and macrophage inflammatory protein 1 alpha  [ **CCL3** ]  and 1 beta.  Ref: 12055257 J Immunol, 2002
- In contrast,  **CCL3** , a ligand of CCR1 and  **CCR5** , induced rapid uptake of FITC dextran by immature DCs but not mature DCs.  Ref: 12609829 Blood< MedlineTA>Blood, 2002
- A number of inflammatory CC chemokines, including MIP 1 alpha  [ **CCL3** ] , MIP 1 beta, RANTES, MCP 2, and HCC 1 9 74 act as  **CCR5**  agonists, while MCP 3 is a natural antagonist of the receptor.  Ref: 12402506 Receptors Channels, 2002
- In EAE protected mice, 17 beta estradiol strongly inhibited mRNA expression of the chemokines RANTES, MIP 1 alpha  [ **CCL3** ] , MIP 2, IP 10, and MCP 1, and of the chemokine receptors CCR1, CCR2 and  **CCR5**  at both time points.  Ref: 11550221 J Neurosci Res, 2001
- The PBMC levels of  **CCR5**  ligands MIP 1 alpha  [ **CCL3** ]  beta and RANTES, and chemokines MCP 1, IL 8, lymphotactin, IP 10 and I 309 were analysed by ribonuclease protection assay RPA.  Ref: 12021948 J Neurol, 2002
- We also determined that  **CCR5**  binding chemokines MIP 1alpha  **CCL3** , CCL4, and RANTES CCL5, specifically by signaling through  **CCR5** , could affect all progenitor B cell populations through a novel mechanism involving heterologous desensitization of CXCR4.  Ref: 12239139 Blood, 2002
- In order to evaluate if these cells are infectabile by human immunodeficiency virus HIV and could be employed as a model in HIV research we exposed these cell lines to X4 T cell tropic and R5 macrophage tropic and subsequently tried to correlate their infectability with i level of chemokine coreceptor CXCR4 and  **CCR5**  expression, ii coreceptor functionality calcium flux, chemotaxis and phosphorylation of MAPK p42 44 and AKT and iii endogenous expression and secretion of HIV related chemokines which compete with the virus for binding to CXCR4 SDF 1 CXCL12 or  **CCR5**  MIP 1beta CCL4, MIP 1alpha  **CCL3** , RANTES CCL5, MCP 2 CCL8, MCP 3 CCL7 and MCP 4 CCL13.  Ref: 11737246 Eur J Haematol, 2001
